# Supplementary material for: Associations of Leisure-Time Physical Activity Trajectories with Fruit and Vegetable Consumption from Childhood to Adulthood: The Cardiovascular Risk in Young Finns Study
Source: Int J Environ Res Public Health. 2019 Nov 12;16(22):4437. doi: 10.3390/ijerph16224437 (PMC6888230; doi:10.3390/ijerph16224437)
Supplement: Supplementary file 1 [file ijerph-16-04437-s001.zip › Supplement3.pdf]

Supplement 3. Filled checklist of guidelines for reporting on latent trajectory studies*.

	**Checklist Item**	**Reported**
1.	Is the metric of time used in the statistical model reported?	Yes
2.	Is information presented about the mean and variance of time within a wave?	No
3a.	Is the missing data mechanism reported?	Yes
3b.	Is a description provided of what variables are related to attrition/missing data?	Yes
3c.	Is a description provided of how missing data in the analyses were dealt with?	Yes
4.	Is information about the distribution of the observed variables included?	Yes
5.	Is the software mentioned?	Yes
6a.	Are alternative specifications of within-class heterogeneity considered (e.g., LGCA vs. LGMM) and clearly documented? If not, was sufficient justification provided as to eliminate certain specifications from consideration?	LGCA nor LGMM were not used. However, LTPA trajectories have been identified from the same data in previous paper by using group-based trajectory modelling.** Those trajectories are in line with the ones identified in this study.
6b.	Are alternative specifications of the between-class differences in variance–covariance matrix structure considered and clearly documented? If not, was sufficient justification provided as to eliminate certain specifications from consideration?	LGCA nor LGMM were not used.

	Checklist Item	Reported
7.	Are alternative shape/functional forms of the trajectories described?	Not applicable in latent profile analyses.
8.	If covariates have been used, can analyses still be replicated?	Yes
9.	Is information reported about the number of random start values and final iterations included?	Yes
10.	Are the model comparison (and selection) tools described from a statistical perspective?	Yes
11.	Are the total number of fitted models reported, including a one-class solution?	Yes
12.	Are the number of cases per class reported for each model (absolute sample size, or proportion)?	Yes
13.	If classification of cases in a trajectory is the goal, is entropy reported?	Yes
14a.	Is a plot included with the estimated mean trajectories of the final solution?	Yes
14b.	Are plots included with the estimated mean trajectories for each model?	No

	Checklist Item	Reported
14c.	Is a plot included of the combination of estimated means of the final model and the observed individual trajectories split out for each latent class?	No
15.	Are characteristics of the final class solution numerically described (i.e., means, *SD/SE*, *n*, CI, etc.)?	Yes
16.	Are the syntax files available (either in the appendix, supplementary materials, or from the authors)?	Yes, from the authors

* See reference: van de Schoot, R., Sijbrandij, M., Winter, S D., Depaoli. S. & Vermunt. J K. The GRoLTS-Checklist: Guidelines for Reporting on Latent Trajectory Studies. Structural Equation Modeling. 2017; 24, 451-467.

** See reference: Rovio, S. et al. Longitudinal physical activity trajectories from childhood to adulthood and their determinants: The Young Finns Study. Scand J Med Sci Sports. 2018; 28, 1073-1083.
