# Supplementary material for: Associations of Leisure-Time Physical Activity Trajectories with Fruit and Vegetable Consumption from Childhood to Adulthood: The Cardiovascular Risk in Young Finns Study
Source: Int J Environ Res Public Health. 2019 Nov 12;16(22):4437. doi: 10.3390/ijerph16224437 (PMC6888230; doi:10.3390/ijerph16224437)
Supplement: Supplementary file 1 [file ijerph-16-04437-s001.zip › Supplement1.pdf]

Supplement 1. Statistical analyses used to identify leisure-time physical activity trajectories and their descriptions.

METHODS: LEISURE-TIME PHYSICAL ACTIVITY TRAJECTORIES

Statistical analysis used to identify leisure-time physical activity trajectories

Latent profile analysis, a type of finite mixture modelling, was conducted separately for males and females to identify distinct LTPA trajectories from childhood to adulthood. Before modelling commenced, the longitudinal data were rearranged so that time points referred to ages instead of measurement years. Because the content of the LTPA questionnaires changed in 1992, overlapping items were recoded for missing values. After recoding, the LTPA questionnaires were similar in content from age 9 to 21 and from age 24 to 48. The latent profile analysis was based on the means of the LTPA outcome measures from age 9 to 48, and error variances were assumed to be equal across classes.

Models with one to six LTPA classes were fitted. Statistical criteria for the goodness-of-fit of each model was used to decide the number of classes. The criteria evaluated in this study were Akaike's information criterion (AIC), the Bayesian information criterion (BIC) and the sample-size adjusted BIC (ABIC). For all three criteria, the lower the value, the better the fit of the model. In addition, the Vuong-Lo-Mendell-Rubin likelihood ratio test (VLMR), Lo-Mendell-Rubin adjusted likelihood ratio test (LMR) and parametric bootstrapped likelihood ratio test (BLRT) statistical tests were used to determine the optimum number of classes. The estimated model was compared to the model with one class less, a lower p-value indicating that the model with one class less is rejected in favour of the estimated model [1]. Entropy values and the average posterior probabilities (AvePP) for most likely latent class membership were used to evaluate the quality of the classification. Both measures ranged from 0 to 1, with the value 1 indicating a perfect classification. Values higher than 0.7 were

considered acceptable for the average posterior probabilities [2, 3]. Missing data were assumed to be missing at random (MAR). Model parameters were estimated by using the full information maximum likelihood (FIML) method with robust standard errors, thereby enabling use of all the available data.

RESULTS: LEISURE-TIME PHYSICAL ACTIVITY TRAJECTORIES

Leisure-time physical activity trajectories of males from childhood to adulthood

The following four LTPA trajectory classes were identified for males: persistently low-active (40.9 %), decreasingly active (15.7 %), increasingly active (31.1 %), and persistently active (12.3 %) (Fig. 1A). At each additional step in the modelling, the ABIC continued to decrease and the p-value of the BLRT remained low (<.001) (Table 2). However, in the five-class solution, the AvePP value for one of the latent classes fell below the acceptable level (<.7), the p-values of the VLMR and LMR were higher than .05 and the BIC started to rise again, hence the four-class solution was considered optimal.

Males on the persistently active trajectory remained the most active (mean LTPA index value 11.6) while those on the persistently low-active trajectory remained the least active almost throughout the study period (mean LTPA index value 7.8). LTPA decreased considerably between ages 12 (mean LTPA index value 9.2; 95% CI 8.9-9.4) and 21 (mean LTPA index value 7.1; 95% CI 6.8-7.3) among the low-active participants. A decline in LTPA for those on the decreasingly active trajectory also begun around age 12 (mean LTPA index value 11.5; 95% CI 11.2-11.8), continuing until age 48 (mean LTPA index value 7.5; 95% CI 6.3-8.7) and thereafter dropping to the level of the low-active participants (mean LTPA index value 7.9; 95% CI 7.4-8.4). At age 18, the LTPA level of the increasers reached its lowest point (mean LTPA index value 8.6; 95% CI 8.3-9.0), and at the age of 24 its highest point (mean LTPA index value 10.3; 95% CI 9.5-11.1) (Fig. 1A).

Leisure-time physical activity trajectories of females from childhood to adulthood

The following five LTPA trajectory classes were identified for females: persistently inactive (16.8 %), persistently low-active (52.4 %), decreasingly active (12.3 %), increasingly active (15.1 %), and persistently active (3.4 %) (Fig. 1B). The p-values of the VLMR and LMR were below .05 only up to the three-class solution. However, since the ABIC continued to decrease substantially and the p-value of the BLRT was lower than .001 in each additional step, the modelling was continued up to the six-class solution (Table 2). At the six-class solution, the AvePP fell below the acceptable level (<0.7) in two of the latent classes, and the BIC started to increase again; hence, the five-class solution was considered optimal.

Among females, the biggest changes in LTPA occurred before age 27, after which it stabilized. The increasingly active trajectory was an exception, showing a continuously increasing trend from childhood to adulthood: the mean LTPA index value increased from 8.5 (95% CI: 8.1-9.0) to 11.2 (95% CI: 9.9-12.5) between ages 9 and 48. The increasers were the second least active participants at age 9, but around age 40 they became the most active, exceeding the LTPA level of the persistently active participants. For those on the decreasingly active trajectory, the decline in LTPA started at age 18 (LTPA index value 10.9; 95% CI: 9.6-12.1) and continued to the end of the follow-up at age 48 (LTPA index value 8.4; 95% CI: 7.3-9.5). The participants on the persistently inactive trajectory remained the least active, showing a slightly decreasing tendency in LTPA across the study period (mean LTPA index value 7.2). A decrease in LTPA was also seen among those on the low-active trajectory from ages 9 (LTPA index value 8.9; 95% CI: 8.6-9.2) to 18 (LTPA index value 7.8; 95% CI: 7.6-8.1), with slight increase in mid-adulthood (Fig. 1B).

Abbreviations

ABIC: Adjusted Bayesian information criterion

AIC: Akaike’s information criterion

AvePP: Average posterior probabilities

BIC: Bayesian information criterion CI: Confidence interval

BLRT: Parametric bootstrapped likelihood ratio test

LMR: Lo-Mendell-Rubin adjusted likelihood ratio test

LTPA: Leisure-time physical activity

VLMR: Vuong-Lo-Mendell-Rubin likelihood ratio test

References

1. Muthén LK, Muthén BO. Mplus User’s Guide (1998-2017). 8th edition. Los Angeles, CA; 2017.

2. Nylund-Gibson K, Choi AY. Ten Frequently Asked Questions about Latent Class Analysis. Transl Issues Psychol Sci. 2018. doi:10.1037/tps0000176.

3. Nagin DS. Group-based modeling of development. Cambridge, Mass.: Harvard University Press; 2005.
